# Supplementary material for: R–R–T (resistance–resilience–transformation) typology reveals differential conservation approaches across ecosystems and time
Source: Commun Biol. 2021 Jan 14;4:39. doi: 10.1038/s42003-020-01556-2 (PMC7809055; doi:10.1038/s42003-020-01556-2)
Supplement: Supplementary file 2 — Supplementary Information [file 42003_2020_1556_MOESM2_ESM.pdf]

Supplementary Material

R-R-T (Resistance-Resilience-Transformation) typology reveals differential conservation approaches across ecosystems and time

Guillaume Peterson St-Laurent[1,2]*, Lauren E. Oakes[2,3,4]*, Molly Cross[2,3] and Shannon Hagerman[1,2]

1. Faculty of Forestry, University of British Columbia, 2900 – 2424 Main Mall, Vancouver, BC, Canada, V6T 1Z4
2. Climate Change Specialist Group, Species Survival Commission, International Union for Conservation of Nature, Rue Mauverney 28, 1196 Gland, Switzerland
3. Wildlife Conservation Society, 1050 East Main Street, Suite 2, Bozeman, MT, USA, 59715
4. Department of Earth System Science, Stanford University, 473 Via Ortega, Stanford, CA, USA 59715

Supplementary Table 1. Overview of CAF projects by year of funding and type of ecosystem. Percentages show proportion out of total number of projects (n=104). Some projects were conducted in more than one ecosystem.

	n	Percentage
**Years of funding**		
2011	6	6%
2012	12	12%
2013	10	10%
2014	13	13%
2015	13	13%
2016	12	12%
2017	12	12%
2018	13	13%
2019	13	13%
**Category of ecosystem**		
Agricultural and grazing lands	11	11%
Coastal aquatic<br>Coastal freshwater/brackish wetlands, estuaries, salt marshes and intertidal	21	20%
Deserts	7	7%
Forests<br>Coniferous, temperate deciduous, mixed-conifer broadleaved and tropical forests	34	33%
Grasslands and savanna<br>Savanna and woodlands, grasslands and scrubland	25	24%
Inland aquatic<br>Inland riparian, floodplain, streams, rivers, lakes, ponds, wetlands, springs, playas and vernal pools	57	55%
Urban/suburban	19	18%
