# Supplementary material for: R–R–T (resistance–resilience–transformation) typology reveals differential conservation approaches across ecosystems and time
Source: Commun Biol. 2021 Jan 14;4:39. doi: 10.1038/s42003-020-01556-2 (PMC7809055; doi:10.1038/s42003-020-01556-2)
Supplement: Supplementary file 3 — Description of Additional Supplementary Files [file 42003_2020_1556_MOESM3_ESM.pdf]

Description of Additional Supplementary Files

File Name: Supplementary Data 1
Description: Short descriptions of CAF projects and R-R-T scores. The descriptions were crafted, on an annual basis, from full proposals by WCS staff to provide a brief abstract of the projects for the general public. They are not always fully representative of the full scope of the CAF projects.
