# Supplementary material for: R–R–T (resistance–resilience–transformation) typology reveals differential conservation approaches across ecosystems and time
Source: Commun Biol. 2021 Jan 14;4:39. doi: 10.1038/s42003-020-01556-2 (PMC7809055; doi:10.1038/s42003-020-01556-2)
Supplement: Supplementary file 4 — Reporting Summary [file 42003_2020_1556_MOESM4_ESM.pdf]

Corresponding author(s): COMMSBIO-20-1681B

Last updated by author(s): Oct 29, 2020

Reporting Summary

Nature Research wishes to improve the reproducibility of the work that we publish. This form provides structure for consistency and transparency in reporting. For further information on Nature Research policies, see our Editorial Policies and the Editorial Policy Checklist.

Statistics

For all statistical analyses, confirm that the following items are present in the figure legend, table legend, main text, or Methods section.

n/a	Confirmed	
☐	☒	The exact sample size (n) for each experimental group/condition, given as a discrete number and unit of measurement
☐	☒	A statement on whether measurements were taken from distinct samples or whether the same sample was measured repeatedly
☐	☒	The statistical test(s) used AND whether they are one- or two-sided<br>*Only common tests should be described solely by name; describe more complex techniques in the Methods section.*
☐	☒	A description of all covariates tested
☐	☒	A description of any assumptions or corrections, such as tests of normality and adjustment for multiple comparisons
☐	☒	A full description of the statistical parameters including central tendency (e.g. means) or other basic estimates (e.g. regression coefficient) AND variation (e.g. standard deviation) or associated estimates of uncertainty (e.g. confidence intervals)
☐	☒	For null hypothesis testing, the test statistic (e.g. F, t, r) with confidence intervals, effect sizes, degrees of freedom and P value noted<br>*Give P values as exact values whenever suitable.*
☒	☐	For Bayesian analysis, information on the choice of priors and Markov chain Monte Carlo settings
☒	☐	For hierarchical and complex designs, identification of the appropriate level for tests and full reporting of outcomes
☒	☐	Estimates of effect sizes (e.g. Cohen's d, Pearson's r), indicating how they were calculated

Our web collection on statistics for biologists contains articles on many of the points above.

Software and code

Policy information about availability of computer code

Data collection	N/A
Data analysis	We used the open source R studio (Version 1.2.1335) to carry out all the statistical analysis. For the Kruskal-Wallis tests, we used the packages "DescTools" to carry out Dunn's post hoc test controlled with Bonferroni adjustment. For the Fisher's exact tests, we used the package "rcompanion" to do the post hoc test adjusted by FDR method for multiple comparisons (Benjamini–Hochberg false discovery rate).

For manuscripts utilizing custom algorithms or software that are central to the research but not yet described in published literature, software must be made available to editors and reviewers. We strongly encourage code deposition in a community repository (e.g. GitHub). See the Nature Research guidelines for submitting code & software for further information.

Data

Policy information about availability of data

All manuscripts must include a data availability statement. This statement should provide the following information, where applicable:

- Accession codes, unique identifiers, or web links for publicly available datasets
- A list of figures that have associated raw data
- A description of any restrictions on data availability

We have conducted our study following the BREB guidelines (Behavioral Research Ethics Board) at the University of British Columbia, Canada (Ethics ID number H19-02949). The dataset used to conduct this study does not include data actively collected by researchers with study participants. The data consists of successful grant proposals submitted by unaffiliated organizations (hereafter "grant partners") to the Wildlife Conservation Society Climate Adaptation Fund between 2011 and 2019. These proposals are owned by the Wildlife Conservation Society, but they contain grant partners' confidential data, and in some cases, intellectual property (e.g., novel technology). These proposals are therefore treated in the same way than other sensitive data collected with human subject. It is BREB's position that a breach of confidentiality of study participants (i.e., grant partners) has taken place when there is a failure to conform to the commitment that the

researchers have made to the study participants when some or all the data has entered the public domain (i.e., the data has become available to any person who is not authorized to view or access the data). Thus, we shall not publicly disclose the raw research data in its original form (e.g., full grant proposals).

Supplementary material 1 provides the year of funding, grant partner organization, type(s) of ecosystems, R-R-T score, title and short description for each of the 104 projects included in the analysis. These descriptions were crafted from full proposals by WCS staff—on an annual basis with a consistent approach—to provide a brief abstract of the projects for the general public; to provide this level of detail for each project, we include this publicly-available description. They are not always completely representative of the full scope of the funded projects, and may thus not always allow replicability of our study. However, if readers are interested in requesting the raw dataset from this publication, they can contact the lead authors, and data can be shared (removing such confidential information such as organization's financial status) upon request. They must sign a non-disclosure agreement and comply with the BREB and the Wildlife Conservation Society's guidelines for further use of the data. Readers may contact loakes@wcs.org if they want to request data.

Field-specific reporting

Please select the one below that is the best fit for your research. If you are not sure, read the appropriate sections before making your selection.

☐ Life sciences ☒ Behavioural & social sciences ☐ Ecological, evolutionary & environmental sciences

For a reference copy of the document with all sections, see nature.com/documents/nr-reporting-summary-flat.pdf

Behavioural & social sciences study design

All studies must disclose on these points even when the disclosure is negative.

Study description

Data collection and analysis draw on methods from the social sciences, but the data analyzed included information specific to ecological and environmental sciences. This specific study does not involve any data collected through interactions with human research participants. However, this study is part of a larger project that involves human research participants. The project as a whole was reviewed and has received ethical approval from the BREB (Behavioral Research Ethics Board) at the University of British Columbia, Canada (Ethics ID number H19-02949). We used a mixed-method approach. During the qualitative portion of our data collection and analysis, we carried out a content analysis of 104 project proposals that were awarded grants from the Climate Adaptation Fund (CAF) between 2011 and 2019 to categorize the projects using our new typology, a six-point continuous interval scale named the RRT scale (for Resistance-Resilience-Transformation). In total, we completed three rounds of coding with a total of four independent coders. We then carried out a quantitative analysis using the R-R-T score assigned to each project during the qualitative portion of our study to explore trends over time and between ecosystems.

Research sample

We used an existing dataset that consists of the proposals of 104 successfully-funded adaptation projects submitted by a total of 88 organizations to the Wildlife Conservation Society (WCS) Climate Adaptation Fund. The 104 projects were implemented across 40 States in the United States between 2011 and 2019. The structure of these proposals changed over the years, but they all include information about (1) the main applicant organization, (2) the targeted region, (3) the climate adaptation challenge(s), (4) the climate-related information and knowledge used to justify/inform the project design, (6) the main objective(s) of the project, (7) the proposed actions to be carried out in order to meet the objective(s), and (8) a plan for communication and engagement.

Sampling strategy

We used an availability sampling strategy by using an existing dataset of a unique portfolio of 104 conservation projects explicitly designed for conservation adaptation. This study offered a unique opportunity to assess a large sample of innovative on-the-ground adaptation initiatives, but it also carried limitations. The portfolio is not a random subsample of adaptation projects conducted in the United States, and as such is not representative of the field of practice, or the broader conservation community.

Data collection

We carried out a content analysis of full proposals for projects that were awarded grants from the Climate Adaptation Fund (CAF) to categorize the portfolio of CAF projects using the qualitative analysis software NVIVO (version 12.6.0) and coding project documents directly. Before starting the content analysis process and after several iterations, we developed the first version of the R-R-T scale in the form of a 5-point continuous interval scale (i.e., initially without the "accelerated transformation" category). We performed three rounds of coding, each time using an updated version of the R-R-T scale. We separated the first round of coding into four phases that facilitated a staggered approach of independent coding, group discussion, and iteration of the definitions through analysis of subsets of the full portfolio. During these phases, three team members (including the first two authors) independently coded the full proposals of CAF projects funded during groupings of two or three years (e.g., 2011 and 2019). Three projects that were initially funded did not complete implementation, but we still included them in our analysis. Across all projects, we coded for funded activities; if any activities were removed between the proposal and granting stage, we did not include those activities in our coding of the project. After each phase, the coders compared their results, discussed any disagreements, and reached consensus on a final score for each project. Throughout the process, the team members revised the R-R-T scale (e.g., refining definitions, examples) and the scores from previous phases.

During the second round of coding, a fourth team member (third author) coded all the CAF projects using the latest iteration of the 5-point R-R-T scale. After cross-referencing and identifying divergence in the scores from rounds one and two, we deliberated and reached consensus on a score for each project. As a result of this coding round, the full research team reviewed the R-R-T scale and added a sixth category to account for an emergent level of transformation, making it a 6-point continuous interval scale. In the third and final round of coding, the two first authors independently coded the projects again with the revised scale, reaching intercoder reliability of 95%. We discussed and agreed upon a score for any projects where discrepancies remained.

Timing

The content analysis was carried out between November 2019 and February 2020. The project proposals were submitted to the Wildlife Conservation Society between 2010 and 2019.

Data exclusions	No data was excluded from the analysis; the proposals of all the funded projects between 2011 and 2019 were included in the content analysis (including three projects that were initially funded, but did not complete implementation).
Non-participation	N/A
Randomization	N/A

Reporting for specific materials, systems and methods

We require information from authors about some types of materials, experimental systems and methods used in many studies. Here, indicate whether each material, system or method listed is relevant to your study. If you are not sure if a list item applies to your research, read the appropriate section before selecting a response.

Materials & experimental systems

n/a	Involved in the study
☒	☐ Antibodies
☒	☐ Eukaryotic cell lines
☒	☐ Palaeontology and archaeology
☒	☐ Animals and other organisms
☒	☐ Human research participants
☒	☐ Clinical data
☒	☐ Dual use research of concern

Methods

n/a	Involved in the study
☒	☐ ChIP-seq
☒	☐ Flow cytometry
☒	☐ MRI-based neuroimaging
